# Antimicrobial activity of Asteraceae species against bacterial pathogens isolated from postmenopausal women

**DOI:** 10.1371/journal.pone.0227023

**Published:** 2020-01-06

**Authors:** Marcela Oliveira Chiavari-Frederico, Lidiane Nunes Barbosa, Isabela Carvalho dos Santos, Gustavo Ratti da Silva, Alanna Fernandes de Castro, Wanessa de Campos Bortolucci, Lorena Neris Barboza, Caio Franco de Araújo Almeida Campos, José Eduardo Gonçalves, Jacqueline Vergutz Menetrier, Ezilda Jacomassi, Zilda Cristiani Gazim, Samantha Wietzikoski, Francislaine Aparecida dos Reis Lívero, Evellyn Claudia Wietzikoski Lovato

**Affiliations:** 1 Laboratory of Preclinical Research of Natural Products, Paranaense University, Umuarama, PR, Brazil; 2 Medicinal Plants and Phytotherapics in Basic Attention, Paranaense University, Umuarama, PR, Brazil; 3 Laboratory of Preventive Veterinary Medicine and Public Health, Paranaense University, Umuarama, PR, Brazil; 4 Animal Sciences with Emphasis on Bioactive Products, Paranaense University, Umuarama, PR, Brazil; 5 Biotechnology Applied to Agriculture, Chemistry Laboratory of Natural Products, Paranaense University, Umuarama, PR, Brazil; 6 Clean Technologies, University Center of Maringa, Maringa, PR, Brazil; 7 Technology and Food Safety and Cesumar Institute of Science, Technology and Innovation – ICETI, University Center of Maringa, Maringa, PR, Brazil; Poznan University of Medical Sciences, POLAND

## Abstract

**Purpose:**

Investigation of the antibacterial action of aqueous extracts of *Bidens sulphurea*, *Bidens pilosa*, and *Tanacetum vulgare*, species of Asteraceae family that are popularly used for the treatment of genito-urinary infection.

**Methods:**

The minimum inhibitory concentration (MIC) and minimal bacterial concentration (MBC) of the extracts against standard strains of *Staphylococcus aureus* (ATCC25923), *Enterococcus faecalis* (ATCC29212), *Escherichia coli* (ATCC25922), and *Pseudomonas aeruginosa* (ATCC27853) and against bacteria that were isolated from cultures of vaginal secretions and urine from menopausal women with a diagnosis of recurrent urinary tract infections (rUTI) were determined by broth microdilution.

**Results:**

The MIC values of the three extracts against Gram-positive and Gram-negative standard bacterial strains ranged from 7.81 to 125.00 mg ml^-1^, and the MBC values ranged from 7.81 to 500.00 mg ml^-1^. However, *B*. *sulphurea* was more efficient. In the urine samples, the three extracts inhibited the growth of coagulase-negative *Staphylococcus* spp., and the *B*. *pilosa* was the most active extract against *E*. *coli* compared with the other ones. For the vaginal secretion samples, no significant differences in the inhibition of coagulase-positive *Staphylococcus* spp. and *P*. *mirabilis* were found among the extracts. *T*. *vulgare* and *B*. *sulphurea* were more effective in inhibiting coagulase-negative *Staphylococcus* spp. compared with *B*. *pilosa*. *E*. *coli* was more susceptible to the *B*. *sulphurea* extract compared with the *B*. *pilosa* and *T*. *vulgare* extracts.

**Conclusion:**

The present results suggested the potential medicinal use of Asteraceae species, especially *B*. *sulphurea*, as therapeutic agents against rUTI-related bacteria.

## 1. Introduction

Urinary tract infections (UTIs) are the most common bacterial infections in women, and their incidence rises in the postmenopausal period mainly because of lower estrogen production [1]. Among the types of UTIs, recurrent urinary tract infections (rUTIs) are one of the most common problems in urology. Recent studies indicated that rUTIs should be considered as different from primary UTIs [2].

Among the main causative microorganisms of rUTIs are aerobic Gram-negative bacteria that are present in the intestinal microbiota, including members of the Enterobacteriaceae family, such as the genera *Escherichia*, *Enterobacter*, *Klebsiella*, *Serratia*, *Proteus*, *Salmonella*, and *Shigella* [3]. In community-acquired UTIs, *Escherichia coli* accounts for approximately 85% of cases. In chronic infections and hospital- or structure-related anomalies of the urinary tract, there is a more equitable distribution of different enterobacteria, with a higher prevalence of UTIs that are caused by *Proteus* spp., *Klebsiella* spp., *Enterobacter* spp., *Pseudomonas* spp., and Gram-positive *Staphylococcus saprophyticus* and *Enterococcus* spp. [4].

The bacterial resistance of microorganisms that are isolated from human urinary infections is well recognized, resulting in a reduction of therapeutic efficacy, making such treatments ineffective and expensive, prolonging the course of the disease, increasing the incidence of complications, and increasing the mortality rate [5]. Thus, the lack of new therapeutic agents to replace those that have become ineffective has necessitated the search to discover new alternatives to treat UTIs more effectively.

Medicinal plant-based antimicrobials for the treatment of UTIs are a vast source of potential medications, such as the Asteraceae family, which comprises nearly 1,600 genera and 23,000 species [6]. Among the main species of this family that are popularly used for the treatment of genito-urinary tract and bacterial infections are *Bidens sulphurea* (Cav.) Sch. Bip., *Bidens pilosa* L., and *Tanacetum vulgare* L. [7–9].

*B*. *sulphurea*, popularly known as yellow cosmos or “cosmo-amarelo,” “picão-grande,” and “aster does México,” is an annual herbaceous species from Mexico, but it is considered invasive and intensely disseminated and naturalized in Brazilian territories. It is traditionally used in Brazil to treat bacterial infections and kidney and bladder inflammation [10–12]. *B*. *pilosa*, popularly known as “picão-preto,” “pica-pica,” and “amor-de-mulher” [7], is a small, annual, erect plant that is native to South Africa and widely distributed throughout the world [7,13]. This species is traditionally used in Brazil for the treatment of bacterial infections, inflammation, and genito-urinary infections [7–9]. *T*. *vulgare*, popularly known as “catinga-de-mulata,” is a perennial native plant that is widespread in Europe and western Asia [14]. It is widely used in Brazilian folk medicine for the treatment of bacterial infections, cystitis, and renal infections [8,14].

Despite the popular use of these species for the treatment of bacterial infections and genito-urinary tract infections, the therapeutic activity of these species has not yet been investigated. The present study evaluated the potential antimicrobial activity of aqueous extracts that were obtained by infusions of these species, as recommended by popular use, against bacteria that were collected from urine samples and vaginal secretions from postmenopausal women with a diagnosis of rUTI.

## 2. Materials and methods

### 2.1. Patient recruitment

Prior to the collection of clinical samples, the study received approval from the Ethics Committee on Research Involving Human Beings of UNIPAR (CAEE no. 90949218.2.0000.0109). The participants provided both verbal and written consent for urine and vaginal secretion collection for research purposes. Women in the postmenopausal period (45–70 years old) with a diagnosis of rUTI (three episodes of UTI in the previous 12 months or two episodes in the last 6 months) were included in the study [15]. Women with structural genetic abnormalities in the genital or urinary systems, genital dystopias greater than Pelvic Organ Prolapse Quantification stage 2 [16], genetic or drug-induced immune deficiency, neurological deficiency that affected urinary tract function, or malignant pelvic disease or women who had already undergone pelvic radiotherapy and used antibiotics in the last 4 weeks at the time of sample collection were excluded from the study [17].

### 2.2. Collection of clinical samples

A total of 15 urine samples and 15 samples of vaginal secretions were collected using sterile containers and swabs that contained Aimes medium with activated charcoal (Transystem^™^, Copan Italia, Brescia, Italy), respectively. The samples were obtained from 15 patients who attended a private clinical routine from May to July 2018. Each participant was given a printed sheet outlining the details of the method to be used for urine collection. It was instructed to wash their hands and clean the genital area with soap and water, discard the first jet and collect the mid-stream urine specimens in a sterile container. Immediately after collection, the samples were sent to the Laboratory of Preventive Veterinary Medicine and Public Health of UNIPAR, Brazil. The samples were transported under ice-cold conditions.

### 2.3. Identification of clinical samples

Urine samples were seeded on plates that contained Mannitol Salt Agar and MacConkey Agar and incubated at 37°C for 24 h to isolate Gram-positive and Gram-negative aerobic bacteria. The swabs were first placed in tubes that contained 3 ml of Brain Heart Infusion (BHI) medium and incubated in an oven at 37°C for 24 h. Afterward, they were seeded on plates according to the procedure for urine samples. Subsequently, macroscopic and microscopic analyses and biochemical tests were performed [18]. Gram-positive, catalase-positive cocci underwent a coagulase assay to classify coagulase-positive and coagulase-negative *Staphylococcus*. The Enterobacteriaceae family was biochemically identified using a set of biochemical tests in the Enterobacteria Kit (NewProv, Paraná, Brazil) according to the manufacturer’s instructions.

### 2.4. Determination of susceptibility to antimicrobials

Antibiotic resistance and susceptibility of the identified organisms were determined using the disc diffusion method [19] with commercially available discs of metronidazole (50 μg/disc), amoxicillin (10 μg/disc), norfloxacin (10 μg/disc), and oxacillin (10 μg/disc). The samples were thawed and added to the culture medium to grow each isolate and incubated. After growth, the bacterial inoculum was padronized on the McFarland 0.5 scale and seeded on Mueller Hinton agar using a sterile swab. After 15 min, the antimicrobial-impregnated disks were incubated at 37°C for 18–24 h. Antibacterial activity was evaluated by measuring the diameter of the growth inhibition zones (in millimeters; including the 6.5 mm disc diameter) for each of the microorganisms. The inhibition zones were measured in triplicate.

### 2.5. Multiple antibiotic resistance index

The multiple antibiotic resistance (MAR) index of each strain was calculated according to the formula of Krumperman (1983) [20]: *a / b*, where *a* is the number of antibiotics to which a particular isolate was resistant, and *b* is the total number of antibiotics tested.

### 2.6. Plant material and extract preparation

Botanical material (*B*. *sulphurea* and *B*. *pilosa*) was obtained from the Medicinal Garden of Paranaense University (UNIPAR; S23°47’55”, W53°18’48”), Umuarama, PR, Brazil. One specimen of each species was registered in the Medicinal Garden of Campus 2 of UNIPAR (no. 131 and 40, respectively). Aerial parts of *T*. *vulgare* were collected in the Municipal Nursery of Saudade do Iguaçu, PR, Brazil (S25°41’30.069”, W52°37’06.207”), and an exsiccate was deposited in the Herbarium of the Federal Technological University of Paraná of Dois Vizinhos, PR, Brazil (no. DVPR006294). All of the species were collected in May 2018. The aqueous extracts of the plants were obtained by infusion as recommended by popular use [12] with minor modifications. The dried and ground vegetable material (100 g, the material was pulverized in a knife mill until granulometry of 850 μm) was subjected to an extraction process by infusing with 1 L of boiling water. Extraction was performed until the extraction medium reached room temperature (24 hours). The residue was separated by filtration, and the supernatant was resuspended in ethanol (1:3 extract/ethanol) for the precipitation of proteins and polysaccharides, obtaining a precipitate and an ethanolic supernatant from the infusion (48 hours of precipitation). After complete removal of the organic solvent by rotavaporation (3 hours/400 mL; 45 °C), the extract was subjected to lyophilization (72 hours; -42 °C). The final yields of the extracts of *B*. *sulphurea*, *B*. *pilosa*, and *T*. *vulgare* were 21.43%, 17.45%, and 23.13%, respectively. The extracts were stored in a freezer until use.

### 2.7. Gas chromatography/mass spectrometry

The chemical constituents of the extract samples were identified using a gas phase chromatograph (Agilent 7890 B) coupled to a mass spectrometer (Agilent 5977 A) equipped with an Agilent HP-5MS UI capillary column (30 m × 0.250 mm × 0.25 μm). For the analysis, an injection volume of 1.0 μl of a solution that was prepared by the dissolution of 20 mg of the *B*. *sulphurea*, *B*. *pilosa*, and *T*. *vulgare* extracts in 1.0 ml of methanol was used. The analytical conditions were the following: 280°C injector temperature operating in spline mode (1:2), 280°C transfer line, and 1 ml min^-1^ carrier gas (helium) flow. The initial column temperature was 80°C (1 min) with a ramp of 2°C min^-1^ until reaching 185°C. The temperature remained at 185°C for 1 min, followed by heating at 9°C min^-1^ until reaching 275°C. The temperature remained at 275°C for 2 min, followed by heating at 25°C min^-1^ to 300°C. The temperature was then held at 300°C for 1 min. The extracts of *B*. *sulphurea*, *B*. *pilosa*, and *T*. *vulgare* underwent electron impact ionization scanning at 70 eV with a 40–600 mass/charge ratio (m/z). The ionization source temperature and quadrupole temperature were 230°C and 150°C, respectively. The compounds were identified by comparing their mass spectra with the NIST 11.0 library and by comparing the retention indices (IRs) that were obtained by the homologous series of n-alkane standards (C7-C28; [21]).

### 2.8. Minimum inhibitory concentration and minimum bactericidal concentration of extracts

The minimum inhibitory concentration (MIC) and minimal bacterial concentration (MBC) of the extracts against standard strains and bacteria that were isolated from cultures of vaginal secretions and urine were determined by the broth microdilution method using Mueller Hinton Broth according to the CLSI [19] with modifications. The vegetal extract was dissolved in Tween 80 (2%) and diluted in culture medium to an initial concentration of 500 mg mL^-1^. Then, serial decimal dilutions (1:2) were prepared by adding culture medium to achieve concentrations ranging to 0.97 mg mL^-1^. Thus, a final volume of 100 μL (culture medium plus extract) was distributed in 96-well plates, as well as controls of culture medium and culture medium with extract and Tween. Bacteria were standardized on the McFarland 0.5 scale and the inoculum adjusted to ~ 10^5^ CFU/mL. The tests were performed in triplicate and the plates incubated at 37 °C for 24 hours. Readings were performed after the addition of 10 μl of 10% diluted 2,3,5-triphenyltetrazolium chloride, followed by incubation at 37°C for 30 min. Bacterial growth was considered when the wells presented any pink tone after incubation [22]. The MIC was the lowest concentration of the extract that inhibited bacterial growth. The MBC was determinate by subculturing 10 μL from the culture of each negative well on Mueller Hinton Agar plates as described above [23].

### 2.9 Statistical analysis

Differences between groups were assessed using analysis of variance (ANOVA), followed by Tukey’s *post hoc* test. Values of *p* < 0.05 were considered statistically significant. The results are expressed as mean ± standard error of the mean (SEM). The statistical analyses were performed using Statistica 13.3 software.

## 3. Results

### 3.1. Effect of antibiotics on bacterial pathogens and their MAR index

Thirty-two bacterial samples were isolated from 15 postmenopausal women who were diagnosed with rUTI, predominantly from urine (10; 31.25%) and vaginal secretions (22; 68.75%). Gram-positive (*Staphylococcus* spp.) and Gram-negative (*Escherichia coli* and *Proteus mirabilis*) bacteria were identified in urine and vaginal secretion samples.

The antibiotic resistance of the identified organisms was determined by the disc diffusion method using antibiotics that are routinely used for the treatment of UTIs. Table 1 shows the percentage of urine samples and vaginal secretions that were antibiotic-resistant. Overall, we observed multidrug-resistant isolates, with a majority from vaginal secretions (48; 57.83%) and urine (18; 51.42%).

**Table 1:** Antibiotic resistance (R) percentage of Gram-positive bacteria (*Staphylococcus* spp) and Gram-negative bacteria (*Escherichia coli*, *Proteus mirabilis*) isolated from urine samples and vaginal secretion of menopausal patients with a diagnosis of genitourinary tract infection.

Antibiotics	Urine samples		Vaginal secretion		Urine samples + Vaginal secretion	
	R	%	R	%	Total (R)	%
**Gram-positive bacteria**						
Amoxicillin	1	20	4	28.57	5	27.77
Metronidazole	5	100	12	85.71	17	94.44
Norfloxacin	1	20	4	28.57	5	27.77
Oxacillin	3	60	9	64.28	12	66.66
**Gram-negative bacteria**						
Amoxicillin	2	40	6	66.66	8	57.14
Metronidazole	5	100	9	100	14	100
Norfloxacin	1	25	4	44.44	5	35.71
Total	18	51.42	48	57.83	66	50.38

The MAR index results indicated that all of the tested genitourinary tract isolates of *Staphylococcus* spp. (19 isolates), *E*. *coli* (11 isolates), and *P*. *mirabilis* (3 isolates) had a very high MAR index (> 0.2; Table 2), indicating that the samples were classified as high risk.

**Table 2:** Multiple antibiotic resistance (MAR) index of bacteria isolated from urine samples and vaginal secretion of menopausal patients with a diagnosis of genitourinary tract infection.

Samples	Gram-positive bacteria		Gram-negative bacteria	
	Urine samples	Vaginal secretion	Urine samples	Vaginal secretion
1	0.5	0.25	*0.33	*0.33
2	-	1.00	-	-
3	-	0.75	-	-
4	-	0.25	*1.00	*1.00
5	-	0.50	-	*1.00
6	-	0.25	-	-
7	1.00	0.75	-	*1.00
8	0.25	0.50	-	-
9	-	1.00	-	-
10	0.50	0.50	•0.33	•0.33
11	0.25	0.50	*0.33	*0.66
12	-	0.50	-	-
13	-	0.50	-	•0.33
14	-	-	*0.66	*0.66
15	-	-	-	*1.00

Gram-positive: *Staphylococcus* spp., Gram-negative: *Escherichia coli, •*Proteus mirabilis*.

MAR was calculated according to the method described by Krumperman [20].

### 3.2. Chemical composition of extracts

Table 3 show the gas chromatography profile and probable chemical composition of the *B*. *pilosa*, *T*. *vulgare*, and *B*. *sulphurea* extracts, respectively. *B*. *pilosa* had β-sitosterol (22.33%, C_29_H_50_O, Mw = 414.39) as the most abundant compound, ethyl iso-allocholate (17.52%, C_26_H_44_O_5_, Mw = 436.31), artemetin (12.84%, C_20_H_20_O_8_, Mw = 388.00), β-carotene (12.51%, C_40_H_56_, Mw = 536.00), followed by betulin (11.18%, C_30_H_50_O_2_, Mw = 442.38), decanoic acid 1,1a,1b,4,4a,5,7a,7b,8,9-decahydro-4a,7b-dihydroxy-3-[hydroxymethyl]-1,1,6,8-tetramethyl-5-oxo-9a-*H*-cyclopropa[3,4]benz[1,2-e]azulene-9,9a-diylester,[1aR (1aα,1bβ,4aβ,7aα,7bα,8α,9β,9aα)] (8.53%, C_40_H_64_O_8_, Mw = 672.00), stigmasterol (4.02%, C_29_H_48_O, Mw = 412.37), 3,4,3',4'-Tetrahydrospirilloxanthin (3.40%, C_42_H_64_O_2_, Mw = 600.49), hexadecanoic acid ethyl ester (2.35%, C_17_H_34_O_2_, Mw = 270.26), methyl linolenate (1.57%, C_19_H_32_O_2_, Mw = 292.24), phytol (1.18%, C_20_H_40_O, Mw = 296.30) and 7,8-Epoxylanostan-11-ol, 3-acetoxy (1.02%, C_32_H_54_O_4_, Mw = 502.40). *T*. *vulgare* had artemetin (15.74%, C_20_H_20_O_8_, Mw = 388.00), verrucarol (15.61%, C_15_H_22_O_4_, Mw = 266.15), phytol (14.03%, C_20_H_40_O, Mw = 269.30), 7,8-Epoxylanostan-11-ol, 3-acetoxy (13.12%, C_32_H_54_O_4_, Mw = 502.40), oleanolic acid (10.19%, C_26_H_44_O_5_, Mw = 456.36), followed by ethyl iso-allocholate (9.31%, C_26_H_44_O_5_, Mw = 436.31), stigmasterol acetate (7.43%, C_31_H_50_O_2_, Mw = 454.38), Ergosterol (6.39%, C_28_H_44_O, Mw = 396.33), hexadecanoic acid methyl ester (3.71%, C_17_H_34_O_2_, Mw = 270.26) and betulin (0.58%, C_30_H_50_O_2_, Mw = 442.38). *B*. *sulphurea* had artemetin (C_20_H_20_O_8_, Mw = 388.00) as the most abundant compound (23.28%), followed by 5-methylheptan-2-amine (19.39%, C_8_H_19_N, Mw = 129.15), β-sitosterol (17.15%, C_29_H_50_O, Mw = 414.39), isohumulone (8.18%, C_21_H_30_O_5_, Mw = 362.00), costunolide (6.54%, C_15_H_20_O_2_, Mw = 232.15), phytol (5.98%, C_20_H_40_O, Mw = 296.30), 7,8-Epoxylanostan-11-ol, 3-acetoxy (5.39%, C_32_H_54_O_4_, Mw = 502.40), hexadecanoic acid ethyl ester (3.90%, C_17_H_34_O_2_, Mw = 270.26), octadecanoic acid methyl ester (1.89%, C_19_H_38_O_2_, Mw = 294.47), ethyl iso-allocholate (1.86%, C_26_H_44_O_5_, Mw = 436.31), tridecanoic acid methyl ester (1.43%, C_17_H_34_O_2_, Mw = 270.26), 9,19-Cyclochloestene-3,7-diol, 4,14-dimethyl-, 3-acetate (1.38%, C_31_H_52_O_3_, Mw = 472.39), oleanolic acid (0.82%, C_26_H_44_O_5_, Mw = 456.36) and palustric acid (0.46%, C_20_H_30_O_2_, Mw = 302.22). After determining the composition of the extracts, we evaluated their bactericidal effectiveness in clinical samples and verified whether their popular use is supported by their ethnobotanical efficacy against UTIs.

**Table 3:** Chemical composition of *Bidens pilosa*, *Tanacetum vulgare* and *Bidens sulphurea* extract.

	^a^Compounds	^b^RI_calc_	Relative area (%)			*m/z*	Strutural formula	^c^MS
			*Bidens pilosa*	*Tanacetum vulgare*	*Bidens sulphurea*			
1	5-methylheptan-2-amine	850	-	-	19.39	129.15	C_8_H_19_N	a, b, c
2	Costunolide	1590	-	-	6.54	232.15	C_15_H_20_O_2_	a, b, c
3	Verrucarol	1599	-	15.61	-	266.15	C_15_H_22_O_4_	a, b, c
4	Hexadecanoic acid ethyl ester	1708	2.35	3.71	3.90	270.26	C_17_H_34_O_2_	a, b, c
5	Tridecanoic acid methyl ester	1781	-	-	1.43	270.26	C_17_H_34_O_2_	a, b, c
6	Methyl linolenate	1901	1.57	-	-	292.24	C_19_H_32_O_2_	a, b, c
8	Octadecanoic acid methyl ester	1918	-	-	1.89	294.47	C_19_H_38_O_2_	a, b, c
9	Phytol	2013	1.18	14.03	5.98	296.30	C_20_H_40_O	a, b, c
10	Palustric acid	2044	-	-	0.46	302.22	C_20_H_30_O_2_	a, b, c
11	Isohumulone	2083	-	-	8.18	362.00	C_21_H_30_O_5_	a, b, c
12	Artemetin	2090	12.84	15.74	23.28	388.00	C_20_H_20_O_8_	a, b, c
13	n.i.		1.06	1,09	0.70			
14	n.i.		0.45	0.97	0.67			
15	Ergosterol	2810	t	6.39	-	396.33	C_28_H_44_O	a, b, c
16	Stigmasterol	2900	4.02	-	-	412.37	C_29_H_48_O	a, b, c
17	β-sitosterol	2928	22.33	-	17.15	414.39	C_29_H_50_O	a, b, c
18	Ethyl iso-allocholate	3018	17.52	9.31	1.86	436.31	C_26_H_44_O_5_	a, b, c
19	Betulin	3074	11.18	0.58	-	442.38	C_30_H_50_O_2_	a, b, c
20	Stigmasterol acetate	3115	-	7.43	-	454.38	C_31_H_50_O_2_	a, b, c
21	n.i.		-	1.01	0.59			
22	Oleanolic acid	3125	-	10.19	0.82	456.36	C_26_H_44_O_5_	a, b, c
23	9,19-Cyclochloestene-3,7-diol, 4,14-dimethyl-, 3-acetate	3220	-	-	1.38	472.39	C_31_H_52_O_3_	a, b, c
24	7,8-Epoxylanostan-11-ol, 3-acetoxy-	3324	1.02	13.12	5.39	502.40	C_32_H_54_O_4_	a, b, c
25	β-carotene	4053	12.51	-	-	536.00	C_40_H_56_	a, b, c
26	3,4,3',4'-Tetrahydrospirilloxanthin	4259	3.40	-	-	600.49	C_42_H_64_O_2_	a, b, c
27	Decanoic acid (1,1a,1b,4,4a,5,7a,7b,8,9-decahydro-4a,7b-dihydroxy-3-[hydroxymethyl]-1,1,6,8-tetramethyl-5-oxo-9a-H-cyclopropa [3,4]benz[1,2-e]azulene-9,9a-diyl ester,[1aR-(1aα,1bβ,4aβ,7aα,7bα,8α,9β,9aα)]	4263	8.53	-	-	672.00	C_40_H_64_O_8_	a, b, c
28	n.i.		-	0.49	0.33	-	-	a, b, c
	**Total identified**		**98.45**	**97.01**	**97.65**			

^a^Compounds listed in order of elution in column HP-5MS;

^b^RI = Identification based on retention index using a homologous series of n-alkane C_7_-C_28_ on Agilent HP-5MS column.

^c^MS = identification based on comparison of mass spectra using with the NIST 11.0 library.

Relative area (%): percentage of the area occupied by the compounds in the chromatogram. n.i. = not identified. t = traces. (-) = without compound.

### 3.3. Antibacterial activity

The MIC values of the *B*. *pilosa*, *T*. *vulgare*, and *B*. *sulphurea* extracts (Table 4) against standard strains of Gram-positive and Gram-negative bacteria ranged from 7.81 to 125.00 mg ml^-1^. The MBC values ranged from 7.81 to >500.00 mg ml^-1^. The one-way ANOVA indicated a significant difference in activity against *S*. *aureus* (*F*_2,6_ = 8.64, *p* < 0.05), *P*. *aeruginosa* (*F*_2,6_ = 7.79, *p* < 0.05), and *E*. *coli* (*F*_2,6_ = 67.00, *p* < 0.001) between the extracts. No significant difference in inhibiting *E*. *faecalis* bacteria was found among the extracts (*F*_2,6_ = 4.19, *p* = 0.07). Tukey’s *post hoc* test showed that the extracts of *B*. *pilosa* (MIC = 13.02 mg ml^-1^) and *B*. *sulphurea* (MIC = 7.81 mg ml^-1^) were the most active against the *S*. *aureus* strain compared with *T*. *vulgare* (MIC = 41.66 mg ml^-1^; *p* < 0.05). *P*. *aeruginosa* was more susceptible to the *B*. *sulphurea* extract (7.81 mg ml^-1^) compared with the other extracts (*p* < 0.05). *E*. *coli* was significantly inhibited (*p* < 0.05) by the extracts of *T*. *vulgare* (52.08 mg ml^-1^) and *B*. *sulphurea* (31.25 mg ml^-1^).

**Table 4:** Antibacterial activities of Gram-positive and Gram-negative bacterial strains, express as minimal inhibitory concentration (MIC, mg mL^-1^) and minimal bactericidal concentration (MBC, mg mL^-1^).

Microorganisms	Source	*Bidens pilosa*		*Tanacetum vulgare*		*Bidens sulphurea*	
		MIC	MBC	MIC	MBC	MIC	MBC
**Gram-positive**							
*S*. *aureus*	ATCC25923	13.02±2.60a	>500.00	41.66±10.41b	125.00	7.81±0a	7.81
*E*. *faecalis*	ATCC29212	26.04±5.21a	500.00	52.08±10.41a	62.50	31.25±0a	31.25
**Gram-negative**							
*P*.*aeruginosa*	ATCC27853	13.01±2.60a	62,50	26.04±5.21a	62.50	7.81±0b	500.00
*E*. *coli*	ATCC25922	125.00±0a	>500.00	52.08±10.41b	500.00	31.25±0b	500.00

Values are expressed as mean ± SEM. The averages followed by equal letters in the same line for MIC did not differ by the Tukey HSD test (p <0.05).

Table 4 shows the results of the *in vitro* screening of the antibacterial activity of the aqueous extracts against bacteria that were isolated from urine and vaginal secretion samples from postmenopausal women. In urine samples for coagulase-negative *Staphylococcus* spp., no significant difference was found between the extracts (*F*_2,12_ = 1.72, *p* = 0.21). The one-way ANOVA indicated a significant difference between the extracts against *E*. *coli* (*F*_2,9_ = 4.21, *p* < 0.05) and *P*. *mirabilis* (*F*_2,6_ = 12.00, *p* <0.01). Tukey’s *post hoc* test showed that the extract of *B*. *pilosa* (58.59 mg ml^-1^) was the most active against *E*. *coli* (*p* < 0.05) compared with *T*. *vulgare* (125.00 mg ml^-1^) and *B*. *sulphurea* (78.12 mg ml^-1^). *B*. *sulphurea* (5.85 mg ml^-1^) promoted better inhibition (*p* < 0.05) of *P*. *mirabilis* compared with the extracts of *T*. *vulgare* (125.00 mg ml^-1^) and *B*. *pilosa* (166.66 mg ml^-1^).

Coagulase-positive and -negative *Staphylococcus* spp. was identified in the samples of vaginal secretions (Table 5). No significant difference was found between the extracts in inhibiting coagulase-positive *Staphylococcus* spp. (*F*_2,3_ = 2.35, *p* = 0.24) and *P*. *mirabilis* (*F*_2,3_ = 6.0745, *p* = 0.08813). A significant difference was found between groups in inhibiting coagulase-negative *Staphylococcus* spp. (*F*_2,30_ = 4.82, *p* < 0.05). Tukey’s *post hoc* test showed that *T*. *vulgare* (8.78 mg ml^-1^) and *B*. *sulphurea* (3.14 mg ml^-1^) were more effective in inhibiting coagulase-negative *Staphylococcus* spp. compared with *B*. *pilosa* (37.63 mg ml^-1^).

**Table 5:** Antibacterial activities of Gram-positive and Gram-negative bacteria isolated from urine samples and vaginal secretion of menopausal patients with a diagnosis of genitourinary tract infection, express as minimal inhibitory concentration (MIC, mg mL^-1^) and minimal bactericidal concentration (MBC, mg mL^-1^).

	*Bidens pilosa*		*Tanacetum vulgare*		*Bidens sulphurea*	
Microorganisms	MIC	MBC	MIC	MBC	MIC	MBC
**Urine Sample**						
*Staphylococcus spp*. Coagulase -	37.88±22.26a	>31.25	18.74±5.29a	>62.50	3.17±1.29a	>15.62
*E*. *coli*	58.59±24.18a	>250.00	125.00±0b	>125.00	78.12±15.62a,b	>62.50
*P*. *mirabilis*	166.66±41.66a	>250.00	125.00±0a	>125.00	5,85±1,95b	7.81
**Vaginal secretion**						
*Staphylococcus spp*. Coagulase +	78.12±46.87a	>31.25	8.78±6.83a	>62.50	2.43±1.46a	>15.62
*Staphylococcus spp*. Coagulase -	37.63±14.30a	>31.25	8.78±2.75b	>62.50	3.14±0.66b	>15.62
*E*. *coli*	102.67±14.80a	>250.00	116.07±8.92a	>125.00	62.50±0b	>62.50
*P*. *mirabilis*	93.75±31.25a	>250.00	62.50±0a	>125.00	5.85±1.95a	>62.50

Values ​​are expressed as mean ± SEM. The averages followed by equal letters in the same line for MIC did not differ by the Tukey HSD test (p <0.05).

With regard to inhibiting *E*. *coli*, the one-way ANOVA showed a significant difference between the extracts (*F*_2,18_ = 7.80, *p* < 0.05). *E*. *coli* was more susceptible to the extract of *B*. *sulphurea* (62.5 mg ml^-1^) compared with the extracts of *B*. *pilosa* (102.67 mg ml^-1^) and *T*. *vulgare* (116.07 mg ml^-1^). The MBC values for the clinical samples ranged from 7.81 to >250.00 mg ml^-1^.

## 4. Discussion

Infections that affect the genito-urinary tract are caused by Gram-positive and Gram-negative bacteria and are common in both young and old women. Estrogen deficiency plays an important role in the development of bacteriuria [24]. These infections are a serious public health problem because they are recurrent in many patients and can lead to severe sequelae, such as sepsis, pyelonephritis, kidney damage, and premature delivery, and multiresistant strains [2,25]. Urinary tract infections also often result in chronic recurrence, resulting in the frequent use of antibiotics or long-term antimicrobial prophylaxis that exposes patients to the consequences of chronic use of these drugs and long-term changes in normal microbiota of the vagina and gastrointestinal tract [26]. Although rUTIs usually are not life-threatening, the high incidence significantly increases healthcare costs and can negatively impact patients’ quality of life [2].

Based on this alarming growth of uropathogens that are resistant to existing drugs and the side effects of antibiotics, new therapeutic agents that are less expensive and have fewer adverse effects need to be developed [24]. Preventing recurrent genito-urinary tract infections and improving patients’ quality of life have been the goals of many research groups [2]. Herbal treatment may be a viable solution for the effective treatment of diseases that are caused by bacteria [27].

Medicinal plants comprise a large variety of small molecules with antibiotic properties, especially terpenoids, glycosides, flavonoids, and polyphenols. Most of these small molecules have poor activity compared with the actions of common antibiotics that are produced by bacteria and fungi. However, despite the less potent effects of vegetal derivatives, many plants can successfully combat infections because of synergistic effects of their different pharmacologically active compounds [28]. In the present study, such synergistic antimicrobial effects of the crude extracts were observed.

Oral infusion preparations of *B*. *pilosa*, *B*. *sulphurea*, and *T*. *vulgare* are popularly used or by seat baths [7,12,14]. The present study evaluated the effects of extracts of these plants, prepared by infusion, against bacterial strains that were isolated from urine and vaginal secretion samples from menopausal women with a diagnosis of UTIs, with the goal of validating their popular use. Such scientific validation is beneficial for patients because the use of infusion preparations of these plants in the form of a seat bath to treat UTIs may be associated with fewer systemic side effects compared with the current antibiotics that are used clinically. Importantly, medicinal plants are considered low-cost options [29], which would facilitate patients’ access to such treatment alternatives for UTIs. Such infections are usually recurrent and present high levels of drug resistance.

In the present study, an elevated MAR index was observed for *Staphylococcus* spp. (0.25 to 1.00), *E*. *coli* (0.33 to 1.00), and *P*. *mirabilis* (0.33) that were isolated from urine and vaginal secretion samples. According to Krumperman [20], a MAR index ≥ 0.2 is observed when isolates are exposed to high-risk sources of human or animal contamination. Interestingly, despite the relatively high resistance indices of the studied samples, the extracts effectively inhibited the growth of coagulase-negative *Staphylococcus* spp. in urine samples and inhibited the growth of both coagulase-positive and -negative *Staphylococcus* spp. in vaginal secretion samples from menopausal women who were diagnosed with UTIs. This effect was more evident for the extract of *B*. *sulphurea*, with intermediate action of *T*. *vulgare*. Coagulase-negative *Staphylococcus* is considered a commensal bacteria in humans, and its role as an etiological agent in various infectious processes has been recognized, especially in urinary infections [30,31].

In addition to the involvement of *Staphylococcus* spp. in the development of UTIs, enterobacteria are one of the main causes of rUTIs [32]. Bactericidal and bacteriostatic effects of *B*. *sulphurea* species were also observed against bacteria, especially *E*. *coli* and *P*. *mirabilis*, that were isolated from urine and vaginal secretion samples from patients with rUTIs. Such an effect is important because UTIs have a tendency to present recurrence or chronicity, and more than 85% of these infections are caused by uropathogenic *E*. *coli* [33].

In addition to exerting antibacterial activity against Gram-negative (*P*. *aeruginosa* and *E*. *coli*) standard strains, a bacteriostatic effect of *B*. *sulphurea* was also observed and with lower intensity in *T*. *vulgare* and *B*. *pilosa* extracts. Bacteriostatic drugs inhibit the growth of bacteria in the environment, and actions of the immune system are necessary to eliminate them [34]. In addition to exerting bacteriostatic effects, the previously reported antioxidant effects of these medicinal plants may also modulate the immune response by increasing interleukin-2, lymphocytes, and T-cells and decreasing lipid peroxidation and prostaglandin synthesis [35].

Previous phytochemical analysis of *B*. *sulphurea* identified phenolic compounds, ferulic acid, caffeic acid and sesquiterpene lactones [36,37]. In *B*. *pilosa*, flavonoids, terpenoids, phenylpropanoids, porphyrins and aliphatic and aromatic compounds are present. [38]. In *T*. *vulgare* phenolic compounds, terpenoid, caffeoylquinic acid, douglanin, ludovicin and β-Thujone can be found [39,40]. The chromatographic analysis of the extracts evaluated in this research indicated that artemetin, a flavonoid with antioxidant effects [41], was a major component of the extracts of *B*. *sulphurea* (21%), *T*. *vulgare* (11%), and *B*. *pilosa* (11%). The antimicrobial activity of artemetin has also been reported in other studies [42,43]. β-sitosterol, a potent antimicrobial phytosterol, was identified in the extracts of *B*. *sulphurea* (15%) and *B*. *pilosa* (6%) but not in the extract of *T*. *vulgare* [44,45]. Additionally, the presence of 5-methylheptan-2-amine, isohumulone, costunolide, tridecanoic acid, and octadecanoic acid methyl ester was only observed in the *B*. *sulphurea* extract. The antimicrobial effects of these compounds are well described in the literature [46–49]. The actions of these compounds explain the higher antimicrobial effect of the *B*. *sulphurea* extract compared with *T*. *vulgare* and *B*. *pilosa*.

## 5. Conclusion

The ethnomedicinal form of *Bidens pilosa*, *B*. *sulphurea* and *Tanacetum vulgare* preparation presented antibacterial activity against standard strains and bacteria that were isolated from cultures of vaginal secretions and urine from menopausal women with a diagnosis of recurrent urinary tract infections. However, *B*. *sulphurea* was more efficient. Thus, these results suggest the potential medicinal use of an Asteraceae species, *B*. *sulphurea*, as a therapeutic agent against bacteria that cause rUTIs.

## Abbreviations

CFU: colony-forming unit
MAR: multiple antibiotic resistance
MBC: minimal bacterial concentration
MIC: minimum inhibitory concentration
rUTIs: urinary tract infections
